# Exploration of the Pressurization Condition for Killing Human Skin Cells and Skin Tumor Cells by High Hydrostatic Pressure

**DOI:** 10.1155/2020/9478789

**Published:** 2020-01-30

**Authors:** Toshihito Mitsui, Naoki Morimoto, Atsushi Mahara, Sharon Claudia Notodihardjo, Tien Minh Le, Maria Chiara Munisso, Mariko Moriyama, Hiroyuki Moriyama, Natsuko Kakudo, Tetsuji Yamaoka, Kenji Kusumoto

**Affiliations:** ^1^Department of Plastic and Reconstructive Surgery, Kansai Medical University, 2-5-1 Shin-Machi, Hirakata City, Osaka 573-1191, Japan; ^2^Department of Plastic and Reconstructive Surgery, Graduate School of Medicine, Kyoto University, Sakyou-Ku Yoshida Konoe-Chou, Kyoto City, Kyoto 606-8501, Japan; ^3^Department of Biomedical Engineering, National Cerebral and Cardiovascular Center Research Institute, 6-1 Kishibe Shin-Machi, Suita City, Osaka 564-8565, Japan; ^4^Pharmaceutical Research and Technology Institute, Kindai University, 3-4-1 Kowakae, Higashiosaka City, Osaka 577-8502, Japan

## Abstract

High hydrostatic pressure (HHP) is a physical method for inactivating cells or tissues without using chemicals such as detergents. We previously reported that HHP at 200 MPa for 10 min was able to inactivate all cells in skin and giant congenital melanocytic nevus (GCMN) without damaging the extracellular matrix. We also reported that HHP at 150 MPa for 10 min was not sufficient to inactivate them completely, while HHP at 200 MPa for 10 min was able to inactivate them completely. We intend to apply HHP to treat malignant skin tumor as the next step; however, the conditions necessary to kill each kind of cell have not been explored. In this work, we have performed a detailed experimental study on the critical pressure and pressurization time using five kinds of human skin cells and skin tumor cells, including keratinocytes (HEKas), dermal fibroblasts (HDFas), adipose tissue-derived stem cells (ASCs), epidermal melanocytes (HEMa-LPs), and malignant melanoma cells (MMs), using pressures between 150 and 200 MPa. We pressurized cells at 150, 160, 170, 180, or 190 MPa for 1 s, 2 min, and 10 min and evaluated the cellular activity using live/dead staining and proliferation assays. The proliferation assay revealed that HEKas were inactivated at a pressure higher than 150 MPa and a time period longer than 2 min, HDFas and MMs were inactivated at a pressure higher than 160 MPa and for 10 min, and ASCs and HEMa-LPs were inactivated at a pressure higher than 150 MPa and for 10 min. However, some HEMa-LPs were observed alive after HHP at 170 MPa for 10 min, so we concluded that HHP at a pressure higher than 180 MPa for 10 min was able to inactivate five kinds of cells completely.

## 1. Introduction

High hydrostatic pressure (HHP) is a safe method of physically inactivating cells or tissues promptly without using chemicals, such as detergents, that is commonly used to prepare decellularized tissues. We previously reported that HHP at 200 MPa for 10 min was able to inactivate all cells in porcine skin, human skin, and human nevus tissue (giant congenital melanocytic nevus (GCMN), representative of human congenital skin tumor) [1–6]. Furthermore, HHP at 200 MPa did not damage the extracellular matrix, although HHP at 1000 MPa damaged and altered the epidermal basement membrane to some degree and prevented the survival of human cultured epidermis (hCE) on the pressurized skin or nevus [1]. In addition, we reported that autologous dermis pressurized at 200 MPa without removing cellular debris showed less contracture after grafting than decellularized allogeneic dermis using a porcine model [2].

At present, our exploratory clinical study to investigate the safety and efficacy of a novel treatment combining autologous nevus tissue inactivated by HHP at 200 MPa and a patient's cultured epidermal autograft (CEA) to reconstruct skin defects after removal is ongoing. Our previous studies indicated that HHP at 150 MPa for 10 min was not sufficient to inactivate cells completely, while HHP at 200 MPa for 10 min was able to inactivate cells completely. In this work, we intend to apply HHP to treat malignant skin tumor; however, the conditions necessary to kill each cell type have not yet been explored. In fact, regarding the death pathway after HHP, it was reported that cells after HHP at 200 MPa died through apoptosis, while those pressurized at >300 MPa died immediately following a necrotic pathway [7, 8]. However, the death pathway taken under our HHP conditions has not yet been explored.

In the present study, we explored the critical pressure and pressurization time using five kinds of human skin cells and skin tumor cells, including keratinocytes (HEKas), dermal fibroblasts (HDFas), adipose tissue derived stem cells (ASCs), epidermal melanocytes (HEMa-LPs), and malignant melanoma cells (MMs). We pressurized cells at 150, 160, 170, 180, or 190 MPa for 1 s, 2 min, and 10 min and evaluated the cellular activity.

## 2. Materials and Methods

### 2.1. Cell Lines and Culture Condition

We purchased and prepared five types of cells: human dermal fibroblasts, adult (HDFas; Catalog number: C0135C; Life Technologies Co., Ltd., Carlsbad, CA, USA), adipose-derived stem cells (ASCs; Lot number: ASC0044; DS Pharma Biomedical Co., Ltd., Osaka, Japan), human epidermal keratinocytes, adult (HEKas; Catalog number: C0055C; Life Technologies Co., Ltd.), human epidermal melanocytes, adult (HEMa-LPs; Catalog number: C0245C, Life Technologies Co., Ltd.), and malignant melanoma cells (MMs; Public Health England Culture Collection, Porton Down, UK).

HDFas, ASCs, and MMs were cultured using Dulbecco's modified Eagle's medium (DMEM; “Nissui”1; Nissui Pharmaceutical Co., Ltd., Tokyo, Japan) containing 10% fetal bovine serum (FBS; Hyclone, Logan, UT, USA) and 1% antibiotic/penicillin and streptomycin solution (MP Biomedicals, LLC, Solon, OH, USA) at 37°C, 95% humidity, and 5% carbon dioxide. HEMa-LPs were cultured in M-254 medium (Life Technologies Co., Ltd.) supplemented with PMA-Free Human Melanocyte Growth Supplement-2 (HMGS-2, Life Technologies Co., Ltd.) at 37°C, 95% humidity, and 5% carbon dioxide. HEKas were cultured in EpiLife® medium (Life Technologies Co., Ltd.) and supplemented with 1% EpiLife® defined growth supplement and 0.1% calcium chloride (CaC1_2_) (Life Technologies Co., Ltd.). The medium of each cell line was changed every 3 or 4 days until confluency, at which point cells were washed with phosphate-buffered saline (PBS(-); Takara Bio Inc., Kusatsu, Japan) and then dissociated using TrypeLE™ Express (Life Technologies Co., Ltd.) and passaged. After 3 to 6 passages, 1 × 10^6^ cells were suspended in 1 ml of CELLBANKER® 1 plus (Nippon Zenyaku Kogyo Co., Ltd., Fukushima, Japan) and cryopreserved until subsequent experiments.

Using these five kinds of cells, we evaluated the pressurization condition that killed each cell type completely using the live/dead assay, morphological observation of the cultured cells, and the water-soluble terzolium salts (WST) assay. We then evaluated the death pathway using an apoptosis assay and transmission electron microscopy (TEM) as described below.

### 2.2. Live/Dead Staining of Cells without Pressurization or after Pressurization

We prepared the live/dead staining working solution (Live/Dead Reduced Biohazard Viability/Cytotoxicity Kit #1; Life Technologies Co., Ltd.) by mixing Component A (SYTO10) and Component B (Ethidium homodimer II (EthD-II)) according to the manufacturer's instructions.

Cryopreserved HEKas, HDFas, ASCs, HEMa-LPs, and MMs were rapidly thawed in a water bath, and 1 × 10^6^ cells each were seeded and cultured on a 10 or 15 cm culture dish with the respective culture medium. After reaching subconfluence, the cells were dissociated using TrypeLE™ Express, and cell suspensions of 1 × 10^6^ cells/ml in the respective culture medium were prepared. A total of 200 *μ*l of each cell suspension was then packed in a plastic bag, and 16 bags were prepared for each kind of cell. One bag of each cell type was preserved at room temperature without pressurization in our safety cabinet (control sample), and the other 15 were pressurized under different conditions.

We prepared 15 kinds of pressurization conditions in this study: pressurization at 150, 160, 170, 180, and 190 MPa and pressurization for 1 s, 2 min, and 10 min, respectively, at each pressure. In brief, each bag was placed in a sample chamber of an isostatic pressurization machine (ECHIGO SEIKA, Co., Ltd., Nagaoka, Japan), and the chamber was filled with tap water. The pressure was increased up to the target pressure of 150, 160, 170, 180, or 190 MPa. The target pressure was then maintained for 1 s, 2 min, or 10 min, after which the pressure was reduced. The suspensions of all groups, including the nonpressurization group (control sample), were then moved to 1.5 ml microtubes (Scientific Specialties, Inc., Lodi, CA, USA) and centrifuged at 200*g* for 5 min. The medium was discarded, and 200 *µ*L of the working solution of live/dead staining was added to resuspend the cells. This suspension was incubated for 15 min in the dark and then centrifuged at 200*g* for another 5 min. The medium was discarded, and the cells were then suspended again using 200 *µ*L of the 0.6% paraformaldehyde solution and incubated for at least 15 min in the dark. After this, fluorescence micrographs were taken using a fluorescence microscope (BZ-9000; Keyence Corp., Osaka, Japan).

### 2.3. Morphological Observation and a Proliferation Assay of the Cells after Pressurization

Cryopreserved HEKas, HDFas, ASCs, HEMa-LPs, and MMs were rapidly thawed, and 1 × 10^6^ cells each were seeded on a 10 or 15 cm culture dish and cultured until reaching subconfluence, as mentioned above. Cells were dissociated using TrypeLE™ Express, and cell suspensions of 1 × 10^5^ cells/ml in the respective culture medium were prepared. A total of 5 ml of each cell suspension was then packed in a plastic bag, and 18 bags were prepared for each kind of cell. Three bags were preserved at room temperature without pressurization in our safety cabinet (control sample), and the other 15 were pressurized under different conditions.

We prepared 15 pressurization conditions, and each bag was pressurized as mentioned above. A 100 *µ*L aliquot of each cell suspension, including the nonpressurization group, was then seeded into the well of a 24-well cell culture plate (CORNING, Inc., Corning, NY, USA) with 1 ml of the respective culture medium. Cells were cultured at 37°C, 95% humidity, and 5% carbon dioxide for 7 days without changing the medium. The cell morphology and attachment were observed at 3 h, 1 day, 3 days, and 7 days after seeding using the inverted microscope (Carl Zeiss Co., Ltd., Oberkochen, Germany).

In addition, the proliferation was evaluated quantitatively using a WST-8 (4-(3-(2-methoxy-4-nitrophenyl)-2-(4-nitrophenyl)-2H-5-tetrazolio)-1, 3-benzene disulfonate sodium salt) assay (Cell Counting Kit-8; Dojindo, Kumamoto, Japan). In brief, a 100 *μ*L aliquot of each cell suspension, either after pressurization or without pressurization, was added to each well (*n* = 36 per group of HEKas, HDFas, ASCs, HEMa-LPs, and MMs, every 4 wells) of a 96-well plate (CORNING, Inc.) with 100 *μ*L of the respective culture medium. The plates were then incubated at 37°C in a humidified atmosphere of 5% CO_2_ for 3 h, 1 day, 3 days, or 7 days without changing the medium. At each evaluation time point, 10 *μ*L of the WST-8 assay reagent was added to each well and incubated at 37°C for 1 h. The plate was then gently shaken, and the absorbance of the medium (*n* = 6 at each point in each group) was determined using a multiplate reader at a wavelength of 450 nm. The absorbance of each medium in the vacant wells (*n* = 6) was also measured, and this absorbance was used as an arbitrary zero point.

### 2.4. The Apoptosis Assay Using Annexin V

Cryopreserved HEKas, HDFas, ASCs, HEMa-LPs, and MMs were rapidly thawed and cultured, and cell suspensions of 1 × 10^6^ cells/ml in the respective culture medium were prepared. A total of 300 *μ*l of each cell suspension was then packed into individual plastic bags, with 16 bags prepared for each kind of cell. One bag of each cell type was preserved at room temperature without pressurization in our safety cabinet (control sample), and the other 15 were pressurized under different conditions, as mentioned above.

After pressurization, 100 *μ*l of each cell suspension, including the nonpressurization group (control sample), was transferred to a 100 *μ*l microtube. Then, 100 *μ*l of Muse Annexin V & Dead Cell solution (Merck Millipore, Darmstadt, Germany) was added to each tube followed by incubation for 20 min at room temperature. The percentages of the cell population that was alive, apoptotic, or dead were analyzed using a Muse Cell Analyzer (Merck Millipore).

### 2.5. TEM of HDFas after Pressurization

Cryopreserved HDFas were thawed and cultured, and a 1 mL suspension containing 1 × 10^6^ HDFas was packed in a plastic bag, with 4 bags prepared in total. One bag was kept at room temperature (control sample), and the other 3 were pressurized at 190 MPa. The pressurization time was 1 s, 2 min, or 10 min. These samples were then fixed with 2% glutaraldehyde, 0.1 M sodium cacodylate, and 1 mM CaCl_2_ at 37°C, pH 7.4, for 30 min and washed 2 times with 0.1 M sodium cacodylate and 0.2 M sucrose at 4°C, pH 7.4, for 10 min. The samples were postfixed with 1% osmium tetroxide, 0.1 M sodium cacodylate and 0.15 M sucrose at 4°C, pH 7.4, for 30 min and then dehydrated with graded ethanol (50, 60, 70, 80, 90, 95, 99, and 100%). The samples were infiltrated in Epon 812 resin and then polymerized at 45°C for 12 h, 55°C for 24 h, and 45°C for 12 h. The specimens were cut into ultrathin sections and observed by TEM (JEM-1400Plus; JEOL Ltd., Tokyo, Japan).

### 2.6. Statistical Analyses

Statistical significance was assessed using the Steel Test. All data are expressed as the mean ± standard deviation. *P* values < 0.05 were considered to be statistically significant.

## 3. Results

### 3.1. Live/Dead Cell Staining without Pressurization and after Pressurization

**Figure 1:**
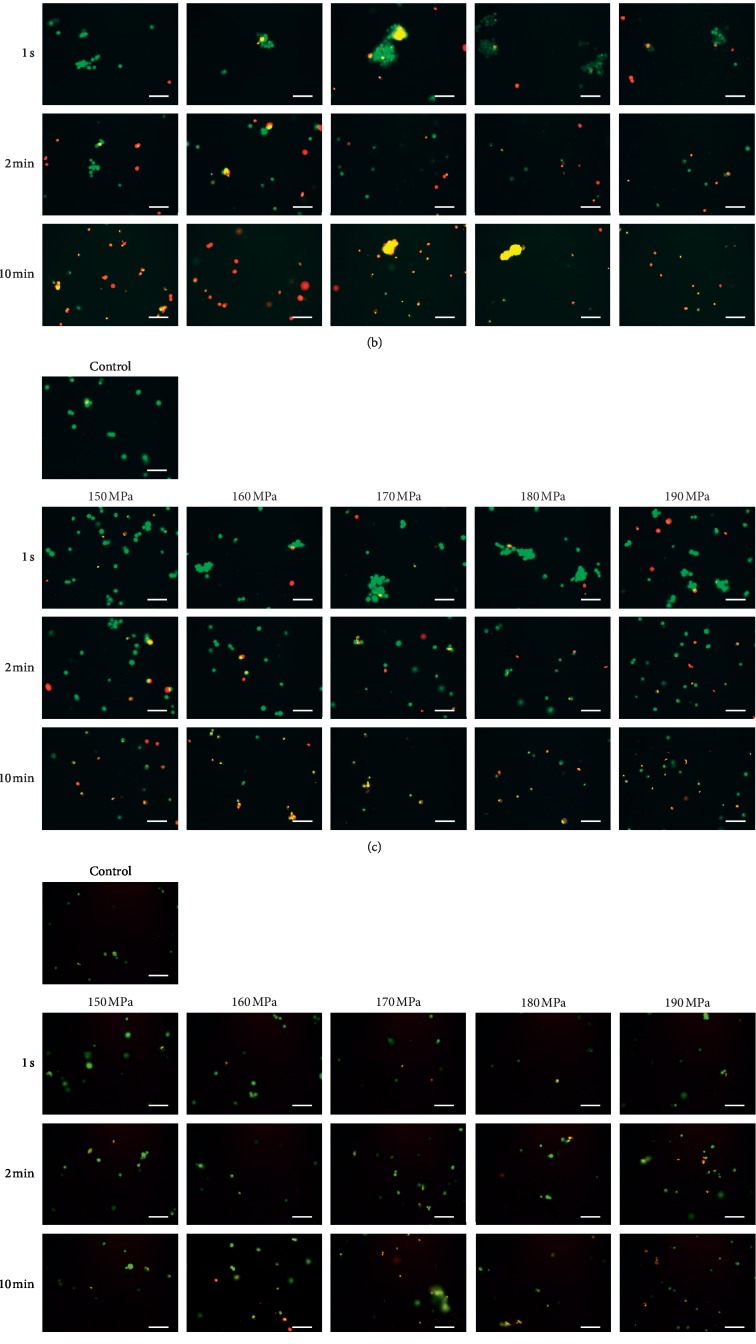
Live/dead staining images of cells after HHP. (a) HEKas, (b) HDFas, (c) ASCs, (d) HEMa-LPs, and (e) MMs. The image of the control group is shown at the upper left in each group. The top row shows the images after HHP at 150, 160, 170, 180, and 190 MPa for 1 s; the middle row shows the images after HHP for 2 min; and the bottom row shows the images after HHP for 10 min. Scale bars = 100 *μ*m.

Regarding the live/dead staining of HEKas, most of the cells without pressurization (control) and with HHP at any pressure for 1 s were stained by the green fluorescence derived from SYTO 10 green fluorescent nucleic acid (Figure 1(a)). The area stained by the red fluorescence derived from ethidium homodimer II nucleic acid stain then increased with HHP at any pressure for 2 min, with most of the cells stained red at 10 min (Figure 1(a)).

Most HDFas without pressurization (control) and with HHP at any pressure for 1 s were stained green (Figure 1(b)). The percentage of HDFas stained red increased with HHP at any pressure for 2 min, and most of the cells were stained red at 10 min (Figure 1(b)).

Most ASCs were stained green in the control sample and at any pressure for 1 s and 2 min. The percentage of ASCs stained red increased with HHP at any pressure for 10 min (Figure 1(c)).

Most HEMa-LPs without pressurization (control) and with HHP at any pressure for 1 s and 2 min were stained green. The percentage of HEMa-LPs stained red increased with HHP at any pressure for 10 min, and most of the cells were stained red above 170 MPa (Figure 1(d)).

Regarding the live/dead staining of MMs, most of the cells were stained green in the control sample and at any pressure for 1 s and 2 min. Most of the cells were stained red at any pressure for 10 min (Figure 1(e)).

### 3.2. Morphological observation of cells without pressurization and after HHP

**Figure 2:**
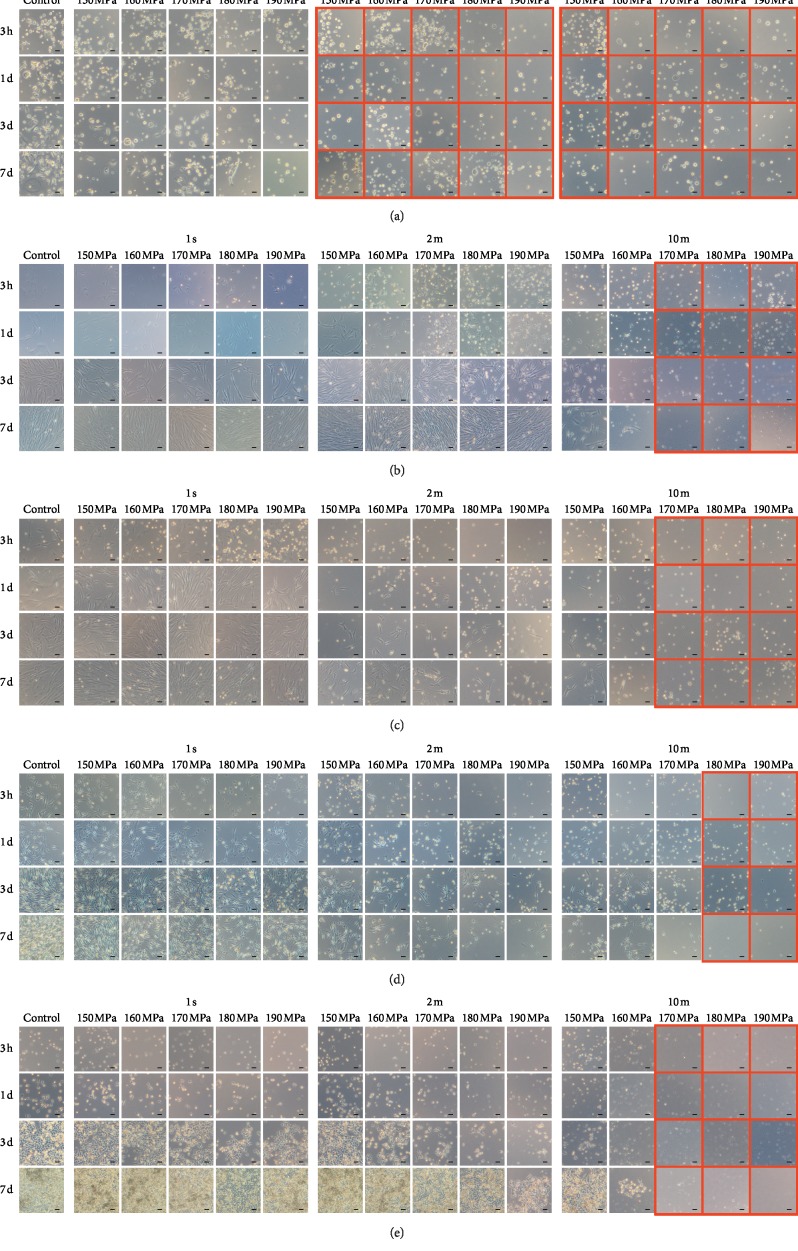
Phase-contrast micrographs of the cells in the control group and after HHP. (a) HEKas, (b) HDFas, (c) ASCs, (d) HEMa-LPs, and (e) MMs. The left column shows the micrographs of cells in the control group at 3 h, 1 day, 3 days, and 7 days after incubation, from top to bottom. The center-left column shows the micrographs of cells after HHP at 150, 160, 170, 180, and 190 MPa for 1 s (left to right) at 3 h 1 day, 3 days, and 7 days after incubation, from top to bottom. The center-right column shows the micrographs of cells after HHP for 2 min, and the right column shows the micrographs after HHP for 10 min. Cells in the red frame are detached and floating. Scale bars = 50 *μ*m.

The micrographs of the cultured cells of the control group (without pressurization) and pressurization groups are shown in Figure 2. All cells in the control group and after HHP at any pressure for 1 s were attached and proliferated for seven days (Figures 2(a)–2(e)). HEKas after HHP at any pressure for 2 and 10 min, indicated by a red frame, were floating, which indicated that they had been inactivated (Figure 2(a)). HDFas and ASCs were floating after HHP at ≥170 MPa (Figures 2(b) and 2(c)), HEMa-LPs were floating after HHP at ≥180 MPa (Figure 2(d)), and MMs were floating after HHP at ≥170 MPa (Figure 2(e)).

### 3.3. Results of the WST-8 Assay of Cells without Pressurization and after HHP

**Figure 3:**
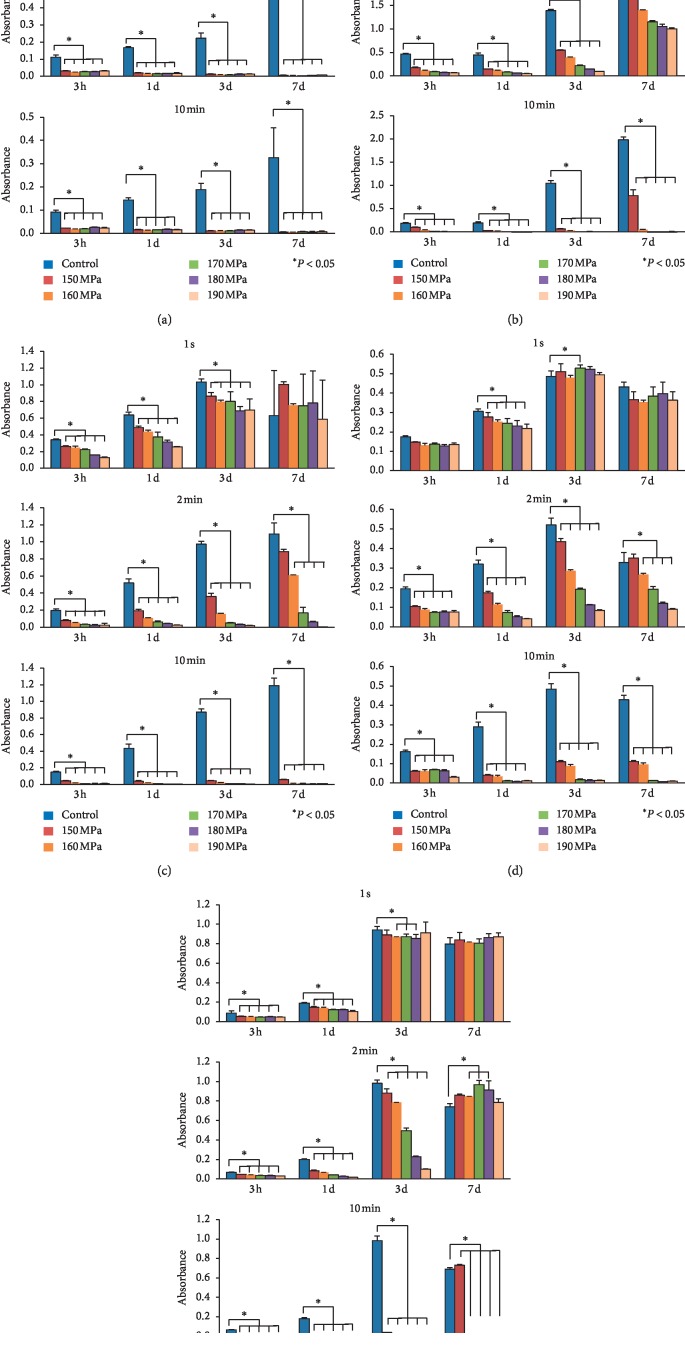
Quantification analyses of cells using the WST-8 assay. (a) HEKas, (b) HDFas, (c) ASCs, (d) HEMa-LPs, and (e) MMs. The top bar chart shows the time course of cell viability in the control group and HHP groups for 1 s. The middle chart shows the time course of cell viability in the control group and HHP group for 2 min. The bottom chart shows the time course of cell viability in the control group and HHP group for 10 min. Statistical significance was assessed using the Steel Test. ^*∗*^*P* values < 0.05 were considered to be statistically significant. ^*∗*^*P* < 0.05 vs. control.

The results of the objective evaluation of cell viability using the WST-8 assay are shown in Figure 3. The growth of HEKas was disturbed after HHP at any pressure for 1 s and completely inhibited after HHP for 2 and 10 min (Figure 3(a)). The growth of HDFas was disturbed after HHP at any pressure for 2 min and completely inhibited after HHP at ≥160 MPa for 10 min (Figure 3(b)). The growth of ASCs was disturbed after HHP at any pressure for 2 min and completely inhibited after HHP at any pressure for 10 min (Figure 3(c)). The growth of HEMa-LPs was disturbed after HHP at any pressure for 2 min and completely inhibited after HHP at any pressure for 10 min (Figure 3(d)). The growth of MMs was disturbed after HHP at any pressure for 2 min for 3 days, but MM cells proliferated at Day 7 (Figure 3(e)); the growth was completely inhibited after HHP at ≥160 MPa for 10 min (Figure 3(e)).

### 3.4. The Apoptosis Assay of Cells Using Annexin V

**Figure 4:**
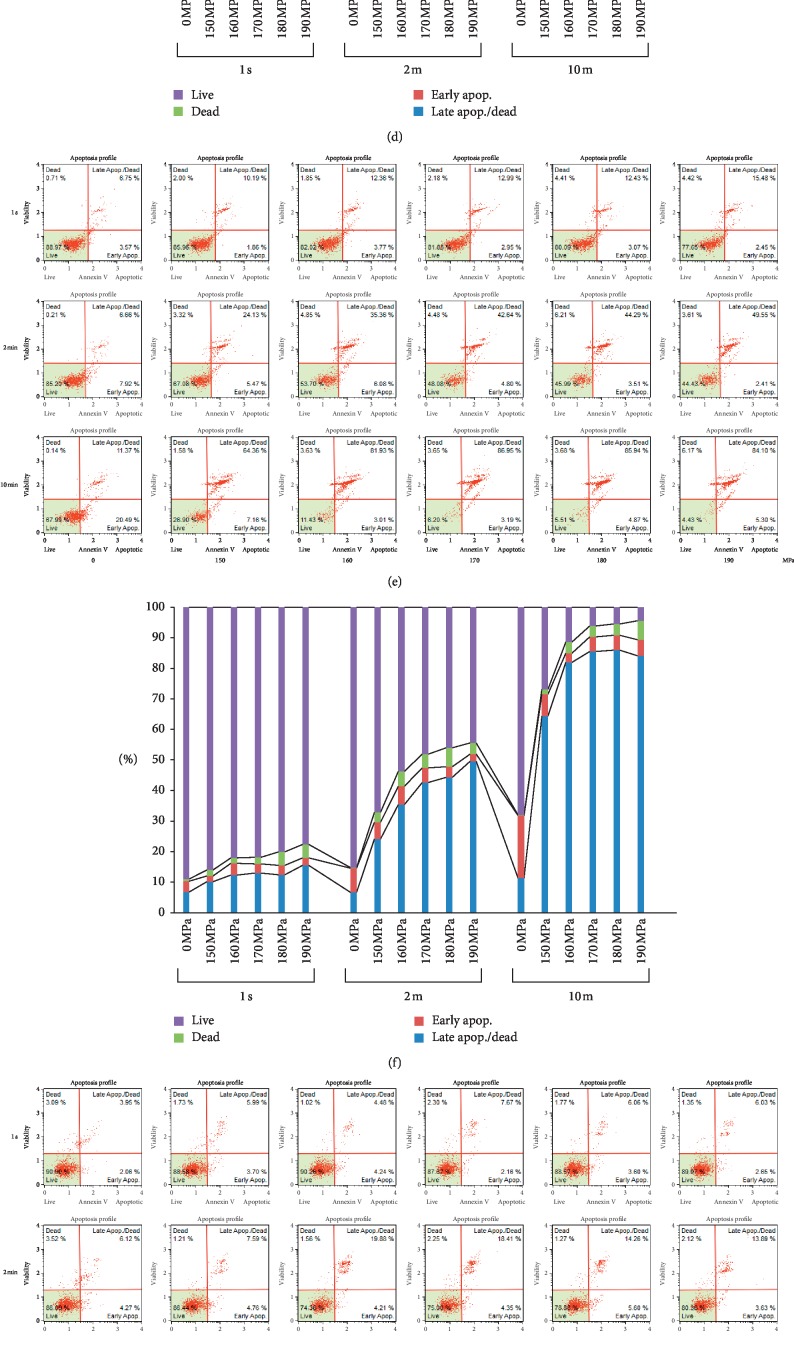
Dot plots and bar charts of the apoptosis assay using Annexin V. (a and b) HEKas, (c and d) HDFas, (e and f) ASCs, (g and h) HEMa-LPs, and (i and j) MMs. The top column shows the dot plot of the cells in the control group (0 MPa) and HHP groups for 1 s. The top left shows the dot plot of the cells in the control group and the 150, 160, 170, 180, and 190 MPa HHP groups from left to right. The middle column shows the dot plot of the cells in the HHP group for 2 min, while the bottom shows the dot plot of the cells in the HHP group for 10 min. The bar chart shows the percentage of live cells, dead cells, early apoptosis cells, and late apoptosis/dead cells of each cell type. The purple bar shows the percentage of live cells. The green bar shows the percentage of dead cells. The red bar shows the percentage of early apoptosis cells. The blue bar shows the percentage of late apoptosis or dead cells.

The dot plots of the apoptosis assay are shown in Figure 4. The vertical axis shows the cell viability as evaluated by the ratio of nonviable cells dyed by 7-Amino actinomycin D (7-AAD). The horizontal axis shows the ratio of the annexin V-positive cells. The lower-left quadrant of the dot plot in which both 7-AAD and Annexin V are negative shows the viable cells, and the lower-right quadrant in which 7-AAD was negative and Annexin V was positive indicates cells in the early stages of apoptosis. The upper-left quadrant in which 7-AAD was positive and Annexin V was negative indicates cells that died through pathways other than the apoptotic pathway, and the upper-right quadrant in which both 7-AAD and Annexin V were positive indicates cells that died by necrosis. Cells that were positive for both 7-AAD and Annexin V in this study were regarded as necrotic cells.

HEKas in the early stages of apoptosis were rare in each condition, and the percentage of necrotic cells increased with increasing pressure and pressurization time, with most HEKas being necrotic after HHP ≥180 MPa for 10 min (Figures 4(a) and 4(b)). Regarding HDFas, there were a small percentage of cells in the early stages of apoptosis at all pressures (Figures 4(c) and 4(d)). The percentage of necrotic cells increased with increasing pressure, and most HDFas were necrotic after HHP at 190 MPa for 10 min (Figures 4(c) and 4(d)). ASCs in the early stages of apoptosis were also rare, and the percentage of necrotic cells increased with increasing pressure and pressurization time, with most ASCs being necrotic after HHP ≥170 MPa for 10 min (Figures 4(e) and 4(f)). Regarding HEMa-LPs, the percentage of viable cells was larger than those of other cells; about 40% of HEMa-LPs were still alive after HHP at 190 MPa for 10 min (Figures 4(g) and 4(h)). Regarding MMs, there were a sizeable percentage of cells in the early stages of apoptosis after HHP for 10 min, and 23% of cells were in the early apoptotic phase after HHP at 190 MPa, with 69% of cells being necrotic (Figures 4(i) and 4(j)).

### 3.5. TEM

**Figure 5:**
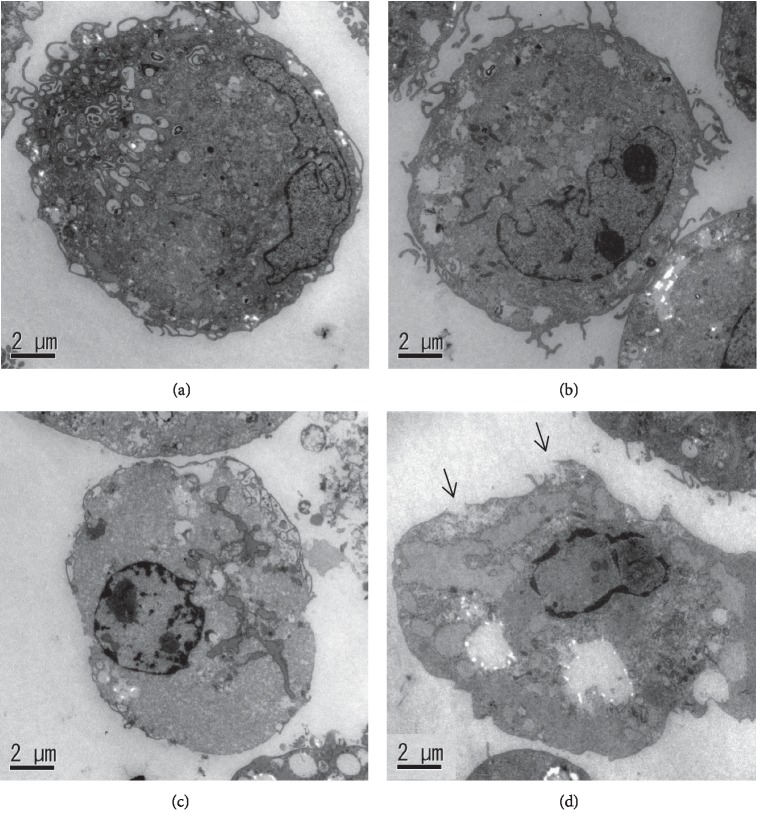
TEM micrographs of HDFas. (a) Without pressurization. (b) After HHP at 190 MPa for 1 s. (c) After HHP at 190 MPa for 2 min. (d) After HHP at 190 MPa for 10 min. Black arrows indicate the rupture of the cell membrane. Scale bars = 2 *μ*m.

The TEM findings of HDFas without HHP and after HHP at 190 MPa are shown (Figure 5). The dendritic structure of the cell membrane was observed in the no-pressurization group (Figure 5(a)) and after HHP for 1 s (Figure 5(b)). In contrast, the structure was missing after HHP for 2 min (Figure 5(c)), and the cell membrane was destroyed for 10 min (Figure 5(d)).

## 4. Discussion

The aim of this study was to explore the critical pressure and pressurization time necessary to inactivate cells related to skin or skin tumor between 150 and 200 MPa after up to 10 min of pressurization. The effect of HHP on human cells was first reported in 1961 [9], and numerous papers have been published concerning the effects of HHP on various kinds of human cells. We reported that human umbilical vein endothelial cells, human aortic smooth muscle cells, and 3T3 cells were completely killed by HHP at 200 MPa for 10 min [10], and we also showed that porcine skin [2, 4, 6] as well as human skin and human nevus tissue were completely inactivated by HHP at 200 MPa for 10 min [1, 3, 5]. Concerning the inactivation of human cells under these pressurization conditions, Peter et al. reported that about 80% of various cell lines were still alive after HHP at 100 MPa for 10 min, and all of the cells were damaged and inactivated after HHP at 350 MPa [11], and Schauwecker et al. showed that HHP at 350 MPa induced devitalization of malignant bone tumor segment [12]. Naal et al. reported that HHP at 200 MPa for 10 min induced irreversible damage of chondral cells [13], and Weiss et al. reported that HHP at 200 MPa for 5 min halted the proliferation of the human adenocarcinoma MCF7 and human Burkitt's lymphoma B-lymphocyte Raji cells [14].

The different types of cell death are often defined by morphological criteria [15]. In fact, we usually focus on the cell growth after culturing, and the morphologic changes in cells to determine the cell viability, as methods for detecting changes in specific enzyme, such as live/dead staining or the apoptosis assay using Annexin V, indicate cell death indirectly. In the present study, the results of the WST-8 assay after culturing and the morphology of the cultured cells showed that HHP at >150 MPa for 2 and 10 min inactivated HEKas, and HHP at >170 MPa for 10 min inactivated HDFas, ASCs and MMs, while HHP at >180 MPa for 10 min inactivated HEMa-LPs. These results suggest that the sensitivity to HHP differs among cell types, but HHP at >180 MPa for 10 min was sufficient to inactivate all types of cells related to GCMN. This supports our previous finding that HHP at 200 MPa for 10 min was able to inactivate all cells in skin samples and GCMN. However, one limitation associated with HHP *in vivo* is that HHP must be used after separating the tissue from the living body, and it cannot inactivate the cells selectively and sterilize the tissue.

Regarding the cell death pathway related to HHP, Takano et al. reported that both necrosis and apoptosis were observed to be induced by pressure in human lymphoblasts [16]. It has also been reported that HHP at 100 MPa for 30 min in erythroleukemia cells and HHP at >150 MPa for 10 min in acute lymphoblastic leukemia cell lines induced apoptosis [17, 18], while HHP at >300 MPa for 5 min in lymphoblast-like human cells induced necrosis [8]. In addition, previous reports have shown that lymphoblast-like human cells died through apoptosis after exposure to 200 MPa for 5 min [8] and that programmed cell death was induced at 150 to 250 MPa for 5 min [19]. The cell death pathways of various cells at various pressures and for a range of pressurization durations have been discussed in detail, and whether apoptosis or necrosis, or both, occurs varies depending on the strength and duration of pressurization, as well as the cell type.

In the present study, HHP at >180 MPa for 10 min inactivated 5 kinds of cells. We considered the main death pathway of these cells to be necrosis, as the death was induced only for 10 min. The Annexin V assay detected that about 20% of MMs showed apoptosis after HHP at 190 MPa for 10 min, suggesting that some percentage of MMs died through apoptosis. However, most of the other types of cells were necrotic, according to the Annexin V assay, and TEM clearly showed the disruption of the cell membrane and swelling of the cells that indicated necrosis due to a damaged cellular membrane. The apoptotic cells detected by the Annexin V assay were negative for 7-AAD and positive for Annexin V, indicating that Annexin V was bound to the phosphatidylserine on the inner surface of the cellular membrane in those cells. This means that this assay was able to detect early apoptotic cells and necrotic cells whose membrane had been disrupted.

Previous reports have shown that the primary cells of porcine skin, human skin, and human nevus tissue were inactivated by HHP at 200 MPa for 10 min [3, 5, 6]. Because the cell death pathway under HHP at 190 MPa for 10 min was mainly necrosis by disruption of the cell membrane, both freshly isolated primary cells and cryopreserved cells would be able to be inactivated under the same pressure and time conditions.

## 5. Conclusions

HHP at ≥180 MPa for 10 min can inactivate various kinds of human cell related to normal skin, furthermore related to malignant skin tumor, such as MMs. These results support our previous finding that HHP at 200 MPa for 10 min was able to inactivate all cells in skin samples and GCMN, which this pressurization condition seems to be applicable to the treatment of malignant skin tumors.

## Data Availability

The data used to support the findings of this study are included in the article.
